# Consequences of spinal cord injury on the sympathetic nervous system

**DOI:** 10.3389/fncel.2023.999253

**Published:** 2023-02-28

**Authors:** Mariah J. Wulf, Veronica J. Tom

**Affiliations:** Marion Murray Spinal Cord Research Center, Department of Neurobiology and Anatomy, Drexel University College of Medicine, Philadelphia, PA, United States

**Keywords:** spinal cord injury, sympathetic nervous system, dysregulation, sympathetic innervation, preganglionic neurons, therapeutics

## Abstract

Spinal cord injury (SCI) damages multiple structures at the lesion site, including ascending, descending, and propriospinal axons; interrupting the conduction of information up and down the spinal cord. Additionally, axons associated with the autonomic nervous system that control involuntary physiological functions course through the spinal cord. Moreover, sympathetic, and parasympathetic preganglionic neurons reside in the spinal cord. Thus, depending on the level of an SCI, autonomic function can be greatly impacted by the trauma resulting in dysfunction of various organs. For example, SCI can lead to dysregulation of a variety of organs, such as the pineal gland, the heart and vasculature, lungs, spleen, kidneys, and bladder. Indeed, it is becoming more apparent that many disorders that negatively affect quality-of-life for SCI individuals have a basis in dysregulation of the sympathetic nervous system. Here, we will review how SCI impacts the sympathetic nervous system and how that negatively impacts target organs that receive sympathetic innervation. A deeper understanding of this may offer potential therapeutic insight into how to improve health and quality-of-life for those living with SCI.

## 1. Introduction

Trauma to the central nervous system (CNS) results in debilitating damage to multiple motor, sensory, and autonomic circuits (Norton and Kobusingye, 2013). Spinal cord injury (SCI) is a devastating form of neurotrauma that is often associated with paralysis but can also result in permanent dysfunction of organs within multiple physiological systems. The pathophysiology of SCI contains two discrete stages: a primary injury and a secondary injury. The primary injury refers to the actual mechanical, tissue damage immediately following injury (reviewed in Alizadeh et al., 2019). There are a variety of different types of primary injuries, e.g., a contusion, a compression, a long-term compression, and a partial or complete transection. Different types of SCI can produce a more severe injury. For example, a complete transection of the spinal cord severs all of the axons within the cord at the level of the injury, leaving no spared tissue. Alternatively, a contusion or a blow to spinal cord often leaves some spared tissue that may be a substrate for functional recovery post-SCI. Therefore, the level and type of injury can greatly influence the amount of dysfunction produced from a SCI. The secondary injury, initiated within minutes of the primary injury event, refers to a multitude of biochemical, cellular, and molecular changes that continue to ensue for weeks to months and further damage the lesion site, and surrounding tissue within the cord, and affected peripheral organs (Alizadeh et al., 2019).

The loss of function that results after SCI is determined by the severity and at which level of spinal segments the injury happened. The higher and more severe the SCI, a larger region of spinal cord is impacted and is no longer connected to the brain. For instance, in the context of sympathetic regulation, a complete SCI above thoracic segment 1 (T1), results in complete loss of modulatory, descending inhibitory supraspinal control over the sympathetic preganglionic neurons (SPNs) located in thoracolumbar spinal cord below the level of injury. This loss of inhibitory control biases the sympathetic system to a more excitatory state. Moreover, intraspinal plasticity of propriospinal and sensory neurons caudal to the injury also influences increased activity of spinal sympathetic neurons and therefore loss of descending inhibition from supraspinal regions along with the intraspinal plasticity both contribute to heightened sympathetic reflexes that culminate in the dysfunction of peripheral organs receiving sympathetic input. On the other hand, a SCI at a lower level (i.e., T11) would disrupt any organ systems receiving sympathetic input below the injury site, but less of the sympathetic system would be “disconnected” from the brain, resulting in fewer organ systems directly impacted by a lower-level SCI than a higher one. Here, we will review the anatomy of the autonomic nervous system, with a focus on the sympathetic nervous system, and how a SCI impacts function of effector organs that are innervated by the sympathetic nervous system.

## 2. Organization of the autonomic nervous system

**FIGURE 1:**
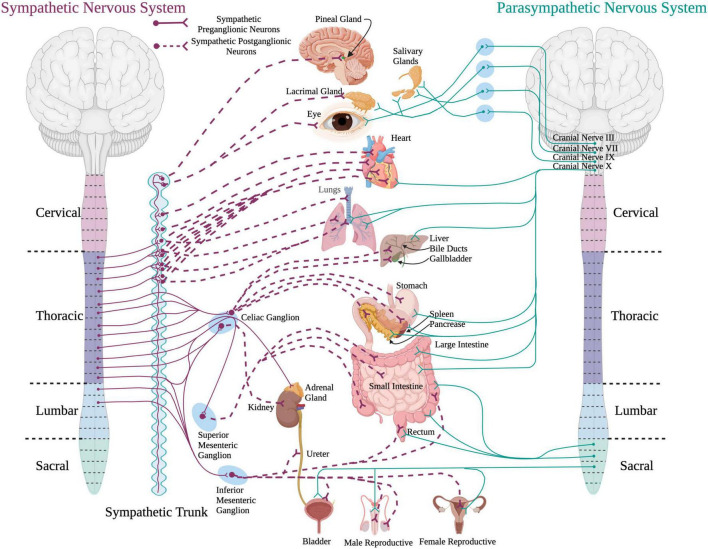
A schematic of the sympathetic and parasympathetic innervation to peripheral organs. Sympathetic preganglionic neurons reside in the intermediolateral cell column in the thoracolumbar spinal cord and project out to and synapse upon sympathetic postganglionic neurons within the sympathetic chain. The sympathetic postganglionic neurons innervate target organs in the periphery. For the parasympathetic system, preganglionic neurons residing in the brainstem and sacral spinal cord send projections directly to target organs in the periphery. (Created using www.biorender.com).

The autonomic nervous system plays an important role in maintaining homeostasis and modulates the involuntary activity of most peripheral organ systems within the body (McCorry, 2007). The sympathetic and the parasympathetic nervous systems are subdivisions of the autonomic nervous system that provide control over multiple peripheral organ systems (Figure 1). The sympathetic nervous system mainly functions during “fight or flight” situations to increase arousal states. The parasympathetic system is involved in “rest and digest” situations to conserve energy for later use.

The SPNs reside in the intermediolateral cell column (IML) of thoracolumbar spinal cord segments T1-L2 (McLachlan and Oldfield, 1981; Oldfield and McLachlan, 1981; Anderson et al., 1989). SPN projections exit the spinal cord *via* the ventral root and release acetylcholine onto sympathetic postganglionic neurons located in ganglia within the sympathetic trunk (also known as the sympathetic chain). From here, sympathetic postganglionic neurons send adrenergic projections to a variety of target organs. Catecholaminergic neurotransmitters, like norepinephrine and epinephrine, are responsible for the “fight or flight” response.

Parasympathetic preganglionic neurons originate within the brainstem and the sacral spinal cord segments (S2–S4). The preganglionic neurons within the brainstem project out *via* cranial nerves III (oculomotor), VII (facial), IX (glossopharyngeal), and X (vagus) (McCorry, 2007; Gibbins, 2013). These parasympathetic preganglionic nerves innervate eye muscles, lacrimal and salivary glands, nasal cavity, and organs in the thorax and abdomen such as the heart, lungs, and digestive system. The parasympathetic preganglionic neurons within the sacral spinal cord exit *via* the pelvic splanchnic nerves to innervate the organs in the lower portion of the body, such as the lower portion of the large intestine, urinary organs, and reproductive organs (McCorry, 2007). Axons of the preganglionic neurons of the parasympathetic system tend to be longer than the axons of the SPNs and project out of the cord to synapse onto parasympathetic ganglia located closer to the target organs compared to the ganglia of the sympathetic nervous system. Pre- and postganglionic neurons of the parasympathetic nervous system release acetylcholine onto effector organs.

Both sympathetic and parasympathetic nervous systems are modulated *via* descending supraspinal control from the brain. Specifically, the paraventricular nucleus of the hypothalamus project directly onto both SPNs and parasympathetic preganglionic neurons. The locus coeruleus, medulla, and raphe nucleus are extra-hypothalamic areas that regulate the sympathetic nervous system. Extra-hypothalamic control over the parasympathetic nervous system includes the amygdala, nucleus ambiguous, raphe nucleus, and the periaqueductal gray. Limbic areas such as the amygdala, cingulate gyrus, and insula also provide descending control by projecting to the hypothalamus that directly innervates both the sympathetic and parasympathetic preganglionic neurons.

## 3. Plasticity of the spinal sympathetic circuit following spinal cord injury

**FIGURE 2:**
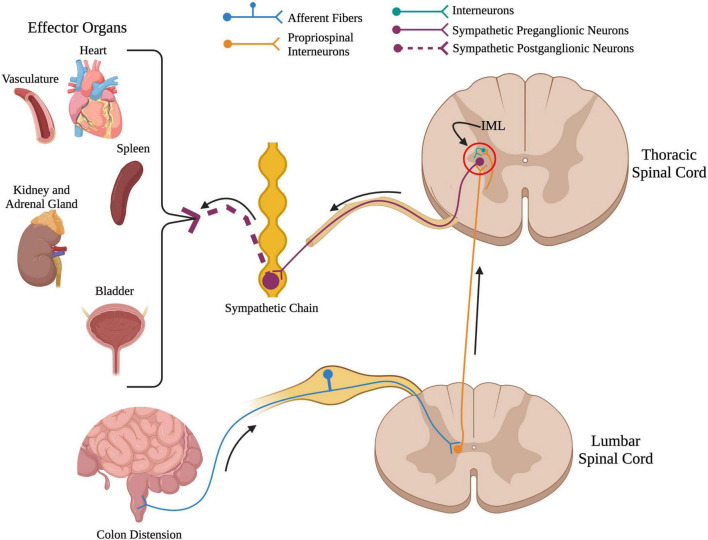
Overview of the spinal sympathetic circuit. When there is a stimuli below the level of injury such as colon distension in the above example, primary afferent fibers from that organ travel into the spinal cord and synapse onto propriospinal interneurons located near the central canal. From here, propriospinal interneurons travel rostrally across many spinal segments to synapse upon either (1) sympathetic preganglionic neurons located in the intermediolateral cell column, or (2) interneurons located near sympathetic preganglionic neurons. Sympathetic preganglionic neurons project out to the sympathetic chain to synapse onto sympathetic postganglionic neurons that innervate target organs in the periphery. (Created using www.biorender.com).

Damage to the spinal cord severs the connections from supraspinal centers to the spinal sympathetic reflex circuit (Figure 2). Because of this, SCI eliminates the feedback regulation of autonomic reflexes and disrupts function of multiple organs and systems that receive sympathetic and parasympathetic input. This is described in more detail below. Not only does SCI sever supraspinal control over the SPNs of the spinal sympathetic circuit, but it also results in neuroplasticity, i.e., changes that affect function of the circuit caudal to the injury site. Following SCI, plasticity occurs at the synapse, cellular, and circuit levels. This intraspinal plasticity within the spinal sympathetic reflex circuit has been shown to contribute to the development of sympathetic hyperreflexia and consequent damage to peripheral organs which increases the risk of mortality in individuals living with SCI.

### 3.1. Primary afferent sprouting

Following SCI, there is sprouting of nociceptive, primary afferent fibers caudal to the injury site, as seen with increased CGRP^+^ (a marker for nociceptive fibers) fibers in the dorsal horn as well as around laminae VII/X around the central canal (Krenz and Weaver, 1998; Hou et al., 2009; Mironets et al., 2018, 2020). This sprouting can be detected as early as 2-weeks post SCI and is thought to be one reason behind increased activation of propriospinal neurons by sensory stimuli described above (Krenz and Weaver, 1998; Hou et al., 2009; Mironets et al., 2018, 2020). In rodent models, this CGRP^+^ fiber sprouting has been shown to be increased within the laminae VII/X around the central canal 8-weeks post injury but not increased within the dorsal horn (Mironets et al., 2020). However, within the human SCI population, CGRP^+^ fiber sprouting has been shown to be present in the chronic stages after SCI (Ackery et al., 2007). Interestingly, increased afferent sprouting has been shown to be dependent on nerve growth factor (NGF) signaling which is also increased following SCI (Bakhit et al., 1991; Brown et al., 2004; Donnelly and Popovich, 2008). This is further supported by the data that shows blocking NGF *via* intrathecal administration of a NGF neutralizing antibody following SCI attenuates this SCI-increased sprouting (Krenz et al., 1999), demonstrating the role of these fibers in sympathetic dysfunction.

### 3.2. Propriospinal and interneuron plasticity

Plasticity of both long-distance propriospinal interneurons as well as local interneurons of the spinal sympathetic circuit has also been implicated in sympathetic dysregulation after SCI. For instance, following SCI, there is sprouting of the primary nociceptive afferents onto long-distance propriospinal neurons, spinal interneurons that projecting across multiple spinal segments (Krenz et al., 1999; Cameron et al., 2006; Hou et al., 2008), including those that project from lumbosacral spinal cord rostrally toward the IML in thoracic spinal cord. As a result, a sensory stimulus below the level of injury could robustly activate more propriospinal interneurons. This increased activation of propriospinal neurons leads to (1) activation of SPNs directly or (2) activation of many local sympathetically correlated interneurons residing in the IML that in turn activate SPNs (Krassioukov et al., 2002; Landrum et al., 2002; Hou et al., 2008; Ueno et al., 2016; Mironets et al., 2018). Local spinal interneurons in the IML in close proximity to SPNs largely contribute most of the input to SPNs. After SCI, it has been shown that a sensory stimulus below the level of injury results in increased activation of this neuron population (Ueno et al., 2016; Mironets et al., 2018). Inhibiting these local excitatory interneurons in the thoracic spinal cord near SPNs effectively diminishes the increased excitability of the spinal sympathetic circuit following injury as well as diminishing SCI-immunosuppression (Ueno et al., 2016; Noble et al., 2022). Moreover, silencing these interneurons delays the onset of SCI-induced plasticity of the sympathetic circuit (Noble et al., 2022). Interestingly, activating excitatory interneurons in uninjured mice partially replicates the anatomical and molecular changes that are seen within the sympathetic circuit following SCI (Noble et al., 2022). Others have reported a decrease of glutamatergic (excitatory) spinal interneurons and an increase of GABAergic (inhibitory) spinal interneuron inputs onto SPNs (Llewellyn-Smith and Weaver, 2001). However, this increase in GABAergic inputs has been suggested to diverge from their canonical, inhibitory function and assume an excitatory role after SCI (Huang et al., 2016). It is apparent that interneuron plasticity is complex and much remains to be understood.

### 3.3. SPN plasticity

Sympathetic preganglionic neurons project out of the spinal cord through the ventral root and depolarize postganglionic neurons that then directly synapse onto target organs. SCI severs the supraspinal descending inhibitory control over SPNs leaving them solely under control of spinal circuits such as spinal interneurons of the sympathetic circuit. This loss of descending inhibition over SPNs leads to decreased inhibition of the spinal sympathetic circuit and, therefore, an exaggerated sympathetic reflex response, i.e., sympathetic hyperreflexia (Ueno et al., 2016; Mironets et al., 2020). SPNs also show profound morphological changes following SCI. For instance, within 3 days post-injury, there is atrophy of SPNs (e.g., decreased soma size and dendrite length) caudal to the injury site (Weaver et al., 1997; Krassioukov et al., 1999; Klimaschewski, 2001; Llewellyn-Smith and Weaver, 2001). However, this atrophy appear to be short-lived and SPNs appear normal morphologically by 2-weeks following SCI (Krassioukov and Weaver, 1996).

### 3.4. Mechanisms that contribute to plasticity of the spinal sympathetic circuit

While elucidating mechanisms that underlie the aforementioned plasticity is still an area of active research, several have been identified. The neuroimmune system is one mechanism that has been proposed to play a role in plasticity in the sympathetic system. Following SCI, there is persistent activation of both central and peripheral neuroimmune and inflammatory processes (Chen et al., 2018). The local inflammatory response post-SCI consists of activation of microglia that reside within the spinal cord and a peripheral immune response consists of infiltrating neutrophils, monocytes, and lymphocytes (T- and B-cells) into the spinal cord. These activated immune cells express factors, such as cytokines [e.g., tumor necrosis factor-alpha (TNFα), IL-1β, and IL-6] and neurotrophic factors (e.g., BDNF, NGF, and BDNF), that have been implicated in driving cellular and anatomical plasticity within the CNS, including within the spinal cord (Donnelly and Popovich, 2008; O’Reilly and Tom, 2020). As mentioned above, sprouting of CGRP^+^ fibers nociceptive primary afferent fibers influences activation of the spinal sympathetic circuit and contributes to sympathetic dysregulation after SCI (Krenz et al., 1999; Cameron et al., 2006; Hou et al., 2008; Mantyh et al., 2011; Mironets et al., 2018). Increased levels of NGF after SCI is associated with sprouting of these fibers that express the NGF-responsive receptor TrkA (Averill et al., 1995; Hou et al., 2008; Mantyh et al., 2011).

Chronically elevated levels of TNFα after SCI have also been shown to contribute to plasticity of the spinal sympathetic circuit. Levels of TNFα are chronically elevated after SCI has also been implicated in sprouting of CGRP^+^ afferent fibers (Mironets et al., 2018, 2020). Inhibiting TNFα signaling *via* the biologic XPro1595 decreases injury-induced sprouting of CGRP^+^ afferent fibers and diminished recruitment of interneurons into the spinal sympathetic circuit. This was associated with diminished autonomic dysreflexia and immune dysfunction associated with sympathetic hyperreflexia (Mironets et al., 2018, 2020).

Another mechanism that was recently identified is SCI-induced expression of thrombospondins (TSP), proteins known to stimulate synapse formation (Liauw et al., 2008; Eroglu et al., 2009; Tyzack et al., 2014; Risher et al., 2018), specifically excitatory synapses, *via* binding to neuronal α2δ-1 calcium channel subunits (Christopherson et al., 2005; Risher and Eroglu, 2012). Following SCI, astrocytes and macrophages increase their secretion of within hours following the initial injury and remain elevated weeks after the injury (Wang et al., 2009; Boroujerdi et al., 2011; Zeng et al., 2013; Brennan et al., 2021). Blocking TSP binding to neuronal α2δ channel subunits after SCI *via* administration of Gabapentin, an anti-epileptic an analgesic drug that also binds to α2δ results in decreased excitatory synaptogenesis and reduces excitability of the sympathetic circuit (Eroglu et al., 2009; Risher and Eroglu, 2012; Risher et al., 2018), demonstrating that TSP plays a role in plasticity within the spinal sympathetic circuit.

Although maladaptive plasticity following SCI contributes to the development of sympathetic hyperreflexia and damage to peripheral organs, it is important to note that plasticity itself is not always detrimental. For example, the spinal bladder reflex circuit and its ability to partially recover following SCI (discussed in more detail below) is a prime example of a potential beneficial effect of SCI-induced plasticity on sympathetic function.

## 4. Dysfunction of organ systems following spinal cord injury

### 4.1. Cardiovascular system

**FIGURE 3:**
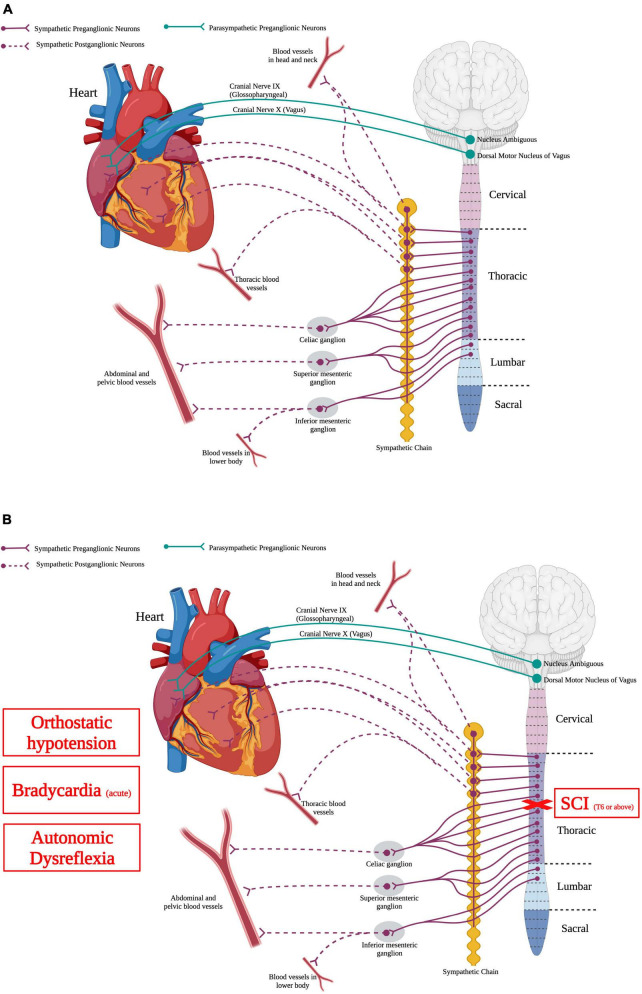
Autonomic control of the cardiovascular system and how SCI impacts the cardiovascular system. **(A)** The heart is innervated by sympathetic postganglionic neurons that receive input from sympathetic preganglionic neurons in thoracic segments 1–4. Parasympathetic regulation of the heart originates from the nucleus ambiguous and dorsal motor nucleus of vagus within the brainstem. Autonomic control over the vasculature is solely from the sympathetic nervous system. **(B)** SCI at or above T6 results in deranged sympathetic activity that results in orthostatic hypotension, bradycardia in the acute phase, and autonomic dysreflexia. (Created using www.biorender.com).

The cardiovascular system delivers oxygen, nutrients, blood, and hormones to sustain peripheral organ systems. In order to do this, the heart pumps blood containing these substances through a complex and vast network of blood vessels present in every type of tissue. Normal cardiovascular function requires a balance of activity between the sympathetic and parasympathetic systems (Figure 3A). Specifically, SPNs from thoracic spinal segment 1 to 4 (T1–T4) send projections to postganglionic neurons in the sympathetic chain that then directly innervate the heart. Moreover, parasympathetic neurons in the brainstem, specifically the dorsal motor nucleus of the vagus and the nucleus ambiguous, send projections out of the cord *via* cranial nerves IX and X and directly innervate the heart (Ciriello and Calaresu, 1982; Hopkins, 1987). While the heart is innervated by both sympathetic and parasympathetic nervous systems, the vasculature is innervated solely by the sympathetic nervous system.

Normally, in uninjured individuals, the sympathetic nervous system releases epinephrine and norepinephrine to accelerate the heart rate and blood pressure while the parasympathetic nervous system reduces heart rate and blood pressure. In times of stress or fearful situations, this increased heart rate *via* sympathetic activation further increases blood flow to the muscles and increases the alertness of the individual. Additionally, when baroreceptors in blood vessels detect a drop in arterial pressure, the sympathetic nervous system is activated and increases vasoconstriction of blood vessels in the periphery. Moreover, increased sympathetic activity has been linked to high blood pressure (Mark, 1996; Schlaich et al., 2004; Grassi, 2009).

Spinal cord injury can disrupt supraspinal regulatory control to the SPNs, producing cardiovascular system dysfunction (Figure 3B). High-level SCI, i.e., at or above thoracic spinal segment 6 (T6), produces hypotension or low resting blood pressure (Mathias et al., 1979; Krassioukov and Claydon, 2006). Orthostatic hypotension occurs when there is a decrease of the systolic blood pressure by 20 mmHg or more, or a decrease in the diastolic blood pressure by 10 mmHg or more (Freeman et al., 2011). Although spinal shock initially following the injury results in hypotension, this hypotensive response can persist for years following SCI (Krassioukov and Claydon, 2006). Cardiac dysrhythmias can also occur after SCI, depending on the level and severity of injury (Krassioukov et al., 2007). These irregular beats of the heart are more serious initially following high-level SCI but seem to diminish as the injury progresses (Krassioukov et al., 2007). Similarly, individuals with high level injuries commonly have cardiac arrhythmias, specifically bradycardia or slowed heartbeat (Mathias, 1976; Winslow et al., 1986; Lehmann et al., 1987). Bradycardia is problematic mainly in the acute phase of SCI (within the first week), which is thought to be due to the initial spinal shock of sudden loss of descending supraspinal input leaving the spinal sympathetic reflex circuit unchecked and an autonomic unbalance between the sympathetic and parasympathetic systems (Frankel et al., 1975; Mathias, 1976; Lehmann et al., 1987; Grigorean et al., 2009; Shaikh et al., 2016).

Autonomic dysreflexia is a life-threatening disorder that is extremely common in the SCI population (Curt et al., 1997). A noxious stimulus below the level of injury, like a full bladder or fecal impaction, causes a massive sympathetic response which leads to vasoconstriction and ultimately hypertension and a concomitant decrease in heart rate (Curt et al., 1997; West et al., 2015; Partida et al., 2016; Mironets et al., 2018, 2020). Loss of descending supraspinal control as well as plasticity within the spinal sympathetic reflex circuit following SCI results in abnormal, heightened sympathetic responses known as sympathetic hyperreflexia. autonomic dysreflexia is a measure used as a readout of sympathetic hyperreflexia as autonomic dysreflexia hallmarks as sudden hypertension and a concomitant decrease in heart rate (Curt et al., 1997; Krassioukov et al., 2003; Zhang et al., 2013). Moreover, such sympathetic hyperreflexia detrimentally impacts other peripheral effector organs. Over time, the number of autonomic dysreflexia events increase in frequency and severity which ultimately increase the individuals’ risk of stroke and myocardial infarction, cardiovascular disease, infection, and death (Krassioukov and Weaver, 1995; Mayorov et al., 2001; Rabchevsky et al., 2012; Zhang et al., 2013; West et al., 2015). Acutely, within the week following SCI, there is early onset of autonomic dysreflexia that is thought to be due to the sudden loss of descending supraspinal input to the spinal sympathetic reflex circuit (Krassioukov et al., 2003; Zhang et al., 2013). However, approximately two weeks post SCI, a second phase of autonomic dysreflexia occurs in which the number of events per day dramatically increase, and the change in blood pressure becomes much more severe (Zhang et al., 2013). This second phase of autonomic dysreflexia is thought to be due to intraspinal plasticity such as sprouting of sensory afferents and propriospinal axons within the spinal sympathetic circuit, leading to sympathetic hyperexcitability as described previously (Cameron et al., 2006; Hou et al., 2008).

Intensification of autonomic dysreflexia over time after SCI has also been associated with detrimental remodeling of the peripheral vasculature (Alan et al., 2010; Rummery et al., 2010; Mironets et al., 2018). After high-level SCI, there is upregulation of adrenergic receptor expression, with results in the vasculature becoming hyperresponsive to vasopressors, e.g., norepinephrine or phenylephrine (Arnold et al., 1995; Landrum et al., 1998; Lee et al., 2016). This remodeling is thought to be a consequence of and also a contributor to the development of autonomic dysreflexia. Furthermore, driving the sympathetic system in rodents with a high-thoracic SCI by repeatedly triggering sympathetic hyperreflexia with colorectal distension results in vasculature that is even more responsive to vasopressors (Alan et al., 2010; Mironets et al., 2018). These data suggest that repeated bouts of sympathetic hyperreflexia exacerbate peripheral cardiovascular dysfunction. Moreover, one could surmise that decreasing the frequency and severity of sympathetic hyperreflexia would diminish detrimental remodeling of the peripheral vasculature. Indeed, treatments that decrease the frequency and severity of sympathetic hyperreflexia after high-level SCI, such as the TNFα inhibitor XPro1595, also attenuate the hyperresponsiveness of peripheral vasculature normally observed after such injuries; arteries from SCI animals treated with XPro1595 have more normal sensitivity to vasopressors than vehicle-treated SCI animals (Mironets et al., 2018).

### 4.2. Pineal gland

The pineal gland is an endocrine gland located deep within the brain that is one of the many organs innervated by the sympathetic nervous system. While melatonin is produced by a variety of organs, the pineal gland is a major source. Melatonin secretion is significantly elevated during the dark phases of circadian rhythms and plays a role in regulating circadian cycles (Lynch, 1971; Yingli Jing and Yan, 2018).

Normally, the amount of light is sensed by the retina, which sends that information to the suprachiasmatic nucleus, which relays that to the paraventricular nucleus (Moore, 1973; Munch et al., 2002). This information then travels down the fibers of the paraventricular nucleus that synapse on SPNs in the IML of the upper thoracic spinal cord segments. The SPNs from the IML project out to the postganglionic neurons located within the cervical sympathetic chain (Bowers et al., 1984). The postganglionic sympathetic neurons directly innervate the pineal gland and release norepinephrine during dark cycles of the circadian rhythm to stimulate melatonin synthesis (Huang and Lin, 1984; Lumsden et al., 2020).

Spinal cord injury can cause significant dysfunction of the pineal gland, depending on the level of injury. An injury in the location of upper thoracic segments or higher would sever the descending fibers of the paraventricular nucleus to the SPNs that regulate function of the pineal gland. An injury in mid-thoracic or lower levels of the spinal cord would not directly impact the fibers descending from the paraventricular nucleus to the SPNs, thus regulation of the pineal gland would remain intact. Accordingly, melatonin levels of individuals with a cervical spinal injury do not increase during the dark cycles of the circadian rhythm, like they would in most able-bodies individuals or individuals with a lower-level SCI (Kneisley et al., 1978; Fatima et al., 2016). These findings support that SCIs below the SPNs that are responsible for projecting out to the postganglionic neurons that innervate the pineal gland result in intact melatonin and pineal gland functioning. This decreased nighttime melatonin levels in SCI individuals may explain dysfunctions of sleep patterns and proper sleep in this population. Increasing levels of melatonin has been explored as a means to treat able-bodied individuals with perturbed sleep patterns. Interestingly, it has been found that following SCI, the pineal gland is able to produce melatonin again with electrical stimulation of the sympathetic pathway responsible for innervating the pineal gland (Lumsden et al., 2020), identifying a possible means to treat sleep disorders after SCI (Scheer et al., 2006).

### 4.3. Visual system

**FIGURE 4:**
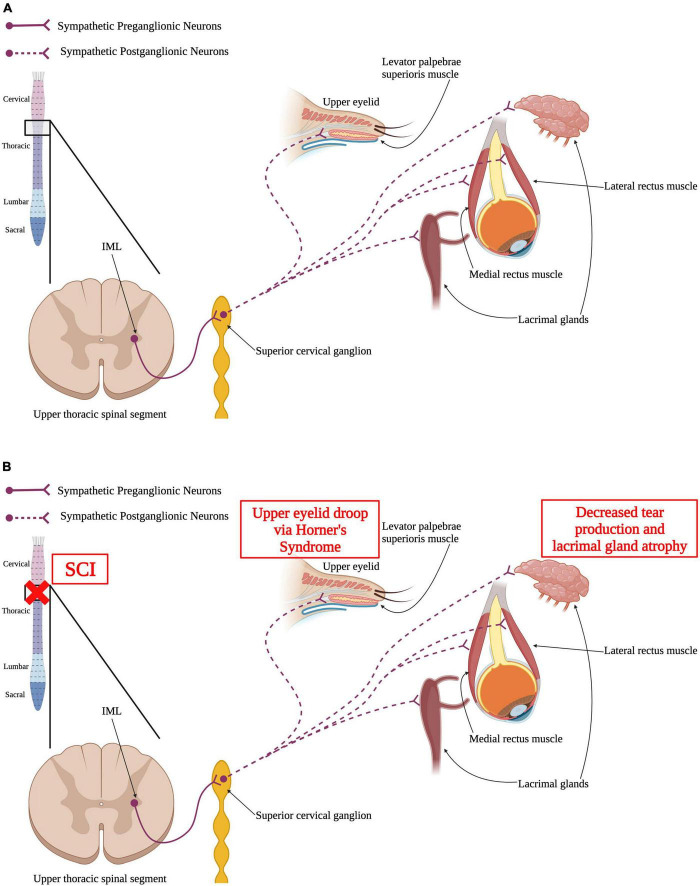
Autonomic control of the visual system and how SCI impacts the visual system. **(A)** Sympathetic preganglionic neurons in upper thoracic spinal segments extend out of the spinal cord *via* the ventral horn to synapse upon sympathetic postganglionic neurons in the superior cervical ganglion. These neurons then directly innervate the levator palpebrae superioris muscle within the upper eyelid, the lacrimal glands, and the medial and lateral rectus muscles. **(B)** SCI may result in Horner’s syndrome where the upper eyelid begins to droop. Similarly, SCI may cause the lacrimal glands to have reduced tear production and atrophy that can ultimately result in dry eye. (Created using www.biorender.com).

Many components of the visual system are also innervated and controlled by the sympathetic nervous system; eye musculature, arteries and nerves around the eyes, and glands associated with the eyes and is ultimately impacted by SCI (Figure 4). The sympathetic pathway innervating the visual system is similar to the one innervating the pineal gland that was previously discussed. SPNs located in the IML of the upper thoracic spinal segments project out to the superior cervical ganglion in the sympathetic trunk, where they synapse on postganglionic neurons (Miller et al., 2005; McDougal and Gamlin, 2015). Those postganglionic neurons project axons to the ciliary ganglion and innervate the ipsilateral eye and surrounding structures, like the lacrimal glands (Tóth et al., 1999; Ruskell, 2003).

**FIGURE 5:**
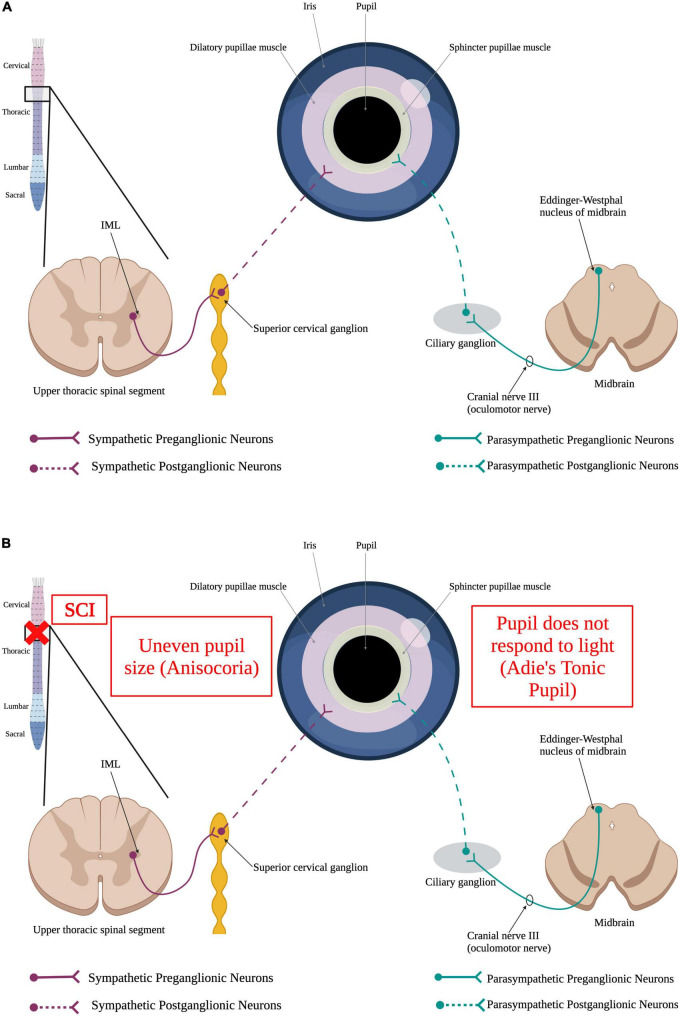
Autonomic control of the pupillary light reflex and how SCI impacts it. **(A)** When the retina detects a dim light, it sends a stimulus to the brain *via* the optic nerve. This signal, in turn, results in the activation of sympathetic preganglionic neurons in the upper thoracic spinal cord that synapse onto sympathetic postganglionic neurons within the superior cervical ganglion. Some of these sympathetic postganglionic neurons innervate the dilatory pupillae muscle (pink). Release of norepinephrine from the sympathetic postganglionic neuron terminals cause the pupil to dilate to allow more light to reach the retina. Alternatively, perception of a bright light by the eye results in the activation of the parasympathetic nervous system. Parasympathetic neurons within Edinger–Westphal nucleus of the midbrain extend their axons out *via* the oculomotor nerve to synapse onto parasympathetic postganglionic neurons within the ciliary ganglion. These postganglionic neurons innervate the sphincter pupillae muscle (yellow) to cause the pupil to constrict, limiting the amount of light reaching the retina. **(B)** SCI causes irregular dilation of pupils resulting in uneven pupil size, a condition known as anisocoria, and Adie’s Tonic Pupil where the pupil does not properly respond to light. (Created using www.biorender.com).

The pupil is a structure of the eye responsible for regulating the amount of light that is allowed to hit the retina. The size of the pupil directly correlates with the amount of light that is able to pass through to the retina. Because of this function, the pupil must be able respond to changing levels of light quickly by contracting or relaxing. This pupillary light reflex is mediated by two muscles, the sphincter pupillae and the dilator pupillae (McDougal and Gamlin, 2015) and is tightly regulated by the autonomic nervous system (Figure 5A). The sphincter pupillae muscle is innervated by the parasympathetic nervous system and the dilatory pupillae is innervated by the sympathetic nervous system (McDougal and Gamlin, 2015). Thus, a SCI in the upper thoracic spinal segments or higher could result in an impaired pupillary light reflex (Figure 5B). Damage to SPNs in the upper thoracic spinal segments can produce Horner’s syndrome, a condition where the pupil is contracted, the upper portion of the eyelid begins to droop, and there is an inability to sweat on one side of the face (Zeitzer et al., 2005; Lee et al., 2007).

Lacrimal glands are located around each eye and are responsible for tear production to supply fluid across the eye to prevent dry eye. Control of lacrimal glands and the production of tears has been shown to be regulated by the sympathetic nervous system (Dartt, 2009; Jin et al., 2020). Following upper thoracic or higher SCI, there is a decrease in tear production and lacrimal gland atrophy (Jin et al., 2020). A decrease in tear production can result in dry eye syndrome that can produce things like ocular surface damage (Dartt, 2004; Stern et al., 2004). Decreases in visual acuity have been shown post-SCI and is thought to be due to the increased risk of hyponatremia within the SCI population compared to able-bodied individuals (Barletta et al., 1994; Giordano et al., 2000; Karlsson and Krassioukov, 2004).

### 4.4. Respiratory system

The respiratory system is made up of the trachea, bronchi, diaphragm lungs, and pulmonary plexus. The main function of the respiratory system is to exchange oxygen for carbon dioxide. As an individual breathes in, oxygen comes into the body and gets transported to peripheral organs in exchange for exporting waste gases, like carbon dioxide. Normal functioning of the respiratory system requires the somatic nervous system to control the muscles involved in breathing. Thus, SCI damage to the somatic nervous system can result in paralysis of the muscles responsible for breathing (e.g., the diaphragm and intercostal muscles), causing respiratory dysfunction. Normal respiratory function also requires autonomic nervous system control over smooth muscles and secretory glands within the respiratory system. The autonomic control requires a balance of both the parasympathetic and sympathetic nervous systems.

Sympathetic preganglionic neurons in the IML of T1–T5 synapse on postganglionic cells in the inferior cervical ganglion that directly innervate the bronchial vasculature and lungs to modulate secretion from submucosal glands (Barnes, 1986; Kummer et al., 1992). The level of injury highly determines the amount of respiratory dysfunction following SCI. An SCI below T5 leaves the sympathetic and parasympathetic innervation of the respiratory tract undamaged. Respiratory complications following high-level SCI is thought to be due to a disrupted balance of the sympathetic and parasympathetic pathways (Ditunno et al., 2004; Berlly and Shem, 2007; Garstang and Miller-Smith, 2007). SCI at or above T5 will sever supraspinal control over the sympathetic system regulating respiratory function but will not impact the parasympathetic system (Krassioukov, 2009).

The parasympathetic and sympathetic nervous systems play opposite roles in control of the smooth muscles within the airways. Specifically, the parasympathetic pathway facilitates contractions of these smooth muscles whereas the sympathetic nervous system facilitates the relaxation of the airway smooth muscles (Grimm et al., 2000; Canning, 2006). Because SCI leads to sympathetic nervous system damage while leaving the parasympathetic system intact, SCI has been shown to disrupt this balance and lead to hyperactivity of the airway smooth muscles which have been associated with increased incidence of asthma within SCI individuals (Grimm et al., 2000; Canning, 2006).

Spinal cord injury patients who experience autonomic dysreflexia have increased risk of pulmonary embolism and edema (Kiker et al., 1982; Aito et al., 2002; Christie et al., 2011; Davison et al., 2012). Pulmonary embolism is when arteries within the respiratory system get blocked *via* blood clots (Aito et al., 2002; Christie et al., 2011). SCI often results in weakness and spasticity of the muscles within the chest, impairing the ability to properly cough to expel foreign objects within the lungs (Roth et al., 1997; Wang et al., 1997). Abnormal autonomic input to the smooth muscles of the airways and increased pulmonary edema have been shown to increase the risk of sleep disordered breathing and sleep-wake disorders within the SCI population (Sankari et al., 2019a,b). Decreased sympathetic regulation to the airways of the respiratory system produces bronchoconstriction and increased nasal resistance which contributes to increased pressure of the upper airway and sleep disordered breathing (Krassioukov, 2009; Wijesuriya et al., 2017). Additionally, as mentioned previously, SCI disruption to the sympathetic nervous system results in impaired melatonin secretion from the pineal gland. This data shows that disruption of circadian rhythms in the SCI population influences the sleep-wake cycles. These findings support that disruptions to the airways as well as to melatonin secretion from the pineal gland can play a role in disrupted sleep patterns and behaviors, preventing individuals from getting necessary sleep.

### 4.5. Liver

The liver is an essential organ that works to metabolize macronutrients, filter blood, and regulate iron storage and release (Goodus and McTigue, 2020). The sympathetic nervous system innervates the liver and plays a role in osmotic homeostasis, metabolism, blood and bile flow, and responses to injury within the liver (Hsu, 1992; Jensen et al., 2013; Kandilis et al., 2015; Mizuno and Ueno, 2017). SPNs located in the IML of T7–12 project out to the celiac ganglion to synapse on postganglionic sympathetic neurons where they form the greater splanchnic nerve and innervate the liver (Hsu, 1992; Yi et al., 2010; Jensen et al., 2013; Mizuno and Ueno, 2017). Therefore, SCI at T7–12 or higher would result in dysfunction of proper hepatic functioning. This overall liver following SCI increases circulating glucose and lipids, as well as insulin resistance which leads to an increased risk of cardiovascular disease, diabetes, and stroke (Bauman and Spungen, 1994, 2001; Farkas and Gater, 2018; Goodus and McTigue, 2020).

Inflammatory responses are the immune system’s way of responding to harmful stimuli and can serve as sort of a defense mechanism for the body. While an acute inflammatory response can be beneficial, if this activated inflammatory response goes unchecked and becomes chronic, such as after SCI, it can start becoming detrimental to peripheral organs such as the liver. The liver is an organ that has been shown to play a strong role in the initiation as well as the progression of the systemic inflammatory response produced by SCI. Immediately following injury, activated macrophages and microglia at the injury site secrete pro-inflammatory cytokines that then enter the blood stream (Hundt et al., 2011; Fleming et al., 2012). The liver detects this increase in proinflammatory cytokines in the blood and begins to produce acute phase response proteins, such as C-reactive protein (CRP), serum amyloid A (SAA), and mannose-binding protein, that work to further increase the inflammatory response in an attempt to minimize the detrimental effects of the injury (Hundt et al., 2011; Fleming et al., 2012). Immediately following SCI and well into the chronic stages, SCI has been shown to increase hepatic leukocyte recruitment as well as increased hepatic proinflammatory cytokines levels (Fleming et al., 2012; Sauerbeck et al., 2015). This data supports that the liver plays a role in the development as well as intensification of the systemic immune response post SCI.

One of the other main functions of the liver is to metabolize macronutrients like fats, proteins, and carbohydrates. When the sympathetic control of the liver is damaged by SCI, altered metabolic activity of the liver ensues. Following SCI, there is increased lipid and ceramide accumulation within the liver the is induced almost immediately following SCI but persists into the chronic phases (Sauerbeck et al., 2015). Ceramides are lipids within the liver that play a role in inflammation and insulin resistance. The SCI-induced increase in ceramides results in inhibition of insulin signaling which further promotes resistance to insulin (Sauerbeck et al., 2015). This increased insulin resistance following SCI is a prominent driver of type 2 diabetes, which is known to be more prevalent within the SCI population (Bauman and Spungen, 1994; Bauman et al., 1999; New et al., 2002; Cragg et al., 2013). Similarly, increased hepatic insulin resistance is also strongly correlated with non-alcoholic fatty liver disease. Therefore, the liver following SCI has shown a similar phenotype to fatty liver disease of non-alcoholic steatohepatitis (Sauerbeck et al., 2015).

With metabolism of nutrients being one of the main functions of the liver, it makes sense that the daily diet of an individual could play a role in attenuating some of the dysfunction of the liver following SCI. Green tea extract has been shown to be hepatoprotective and attenuate hepatic steatosis (Bruno et al., 2008; Park et al., 2011; Masterjohn and Bruno, 2012). Administering a diet rich in green tea extract prior to SCI significantly reduced the amount of iron accumulation associated with the chronic phase of liver disfunction following SCI. However, it did not attenuate liver macrophage activation nor lipid and fatty acid accumulation following SCI (Goodus et al., 2018).

Spinal cord injury-induced damage to the liver has also been associated with diabetes and cardiovascular disease (Bauman and Spungen, 2001; Lee et al., 2005). Low-density cholesterol is directly transported to the arteries and its build up there increases the risk for cardiovascular disease. On the other hand, high-density cholesterol gets transported to the liver, where it can be removed from the blood. SCI has been shown to increase the amount of low-density cholesterol and decrease the amount of high-density cholesterol. High-sensitivity C-reactive protein (hsCRP), one of the acute phase proteins produced by the liver in response to a systemic inflammatory response and has been used as a marker for increased risk of cardiovascular disease, is elevated in SCI patients, indicating that liver dysfunction following SCI is likely to contribute to other metabolic disorders (Lee et al., 2005).

### 4.6. Gallbladder

The gallbladder sits right underneath the liver and is responsible for storing the bile produced by the liver. Bile, a mixture of cholesterol, bilirubin, and bile salts, is released when food is consumed to aid in digestion of fats. The gallbladder is connected to the liver and duodenum of the small intestine through the biliary tract. When uninjured individuals consume food, the parasympathetic nervous system, along with the hormone cholecystokinin produced in the upper regions of the intestine, signals the gallbladder to contract, and release the stored bile into the small intestine to assist in digestion.

The sympathetic nervous system is involved in inhibiting gallbladder contraction (Persson, 1972, 1973; Banfield, 1975). SPNs residing in the IML of T7–10 project axons out *via* the greater splanchnic nerve and synapse upon postganglionic sympathetic neurons in celiac ganglion that innervate the gallbladder and bile ducts (Tandon et al., 1997). SCI at or above T7–T10 severs supraspinal, descending inhibition of the sympathetic nervous system responsible for innervating the gallbladder, thereby reducing the motility of the gallbladder and resulting in stasis (Nino-Murcia et al., 1990).

This reduced motility after SCI increases the likelihood for the development of gallstones, or cholelithiasis, which are hardened stones of bile that are mostly made up of cholesterol that can get lodged within the biliary ducts (Apstein and Dalecki-Chipperfield, 1987; Fong et al., 2003; Baltas et al., 2009). Gallstones can block the cystic duct and cause inflammation of the gallbladder, known as cholecystitis. Consequently, gallstone disease is significantly more common in patients with SCI compared to uninjured patients which greatly increases the risk of acute pancreatitis or inflammation of the pancreas (Apstein and Dalecki-Chipperfield, 1987). The leading mechanism for increased risk of gallstone disease within the SCI population is decreased gallbladder motility that causes a state of inactivity of the gallbladder therefore increasing the risk of gallstone development (Apstein and Dalecki-Chipperfield, 1987; Fong et al., 2003). This less frequent contraction of the gallbladder results in impaired filling and ejection of bile (Nino-Murcia et al., 1990; Fong et al., 2003). This can increase the susceptibility of solidification of bile, also known as bile sludge. With decreased gallbladder motility in SCI individuals, it increases the development of biliary sludge compared to individuals with SCI below T10 (Tandon et al., 1997; Baltas et al., 2009).

### 4.7. Pancreas

The pancreas is an organ that is closely related to the gallbladder. While the gallbladder stores bile produced by the liver, the pancreas produces and stores its own enzymes that assist in digestion and breakdown of food. The pancreas can be broken down into two parts: the exocrine pancreas and the endocrine pancreas. Most of the pancreas is considered the exocrine pancreas that is responsible for producing the pancreatic enzymes to aid in digestion. The endocrine pancreas is comprised of clusters of cells called islet beta cells that work to release hormones, like insulin and glucagon, and works to maintain glucose homeostasis. Both the parasympathetic and sympathetic nervous systems innervate the pancreas. Parasympathetic activity stimulates islet beta cells to secrete insulin whereas sympathetic activity inhibits insulin secretion from the pancreas (Kiba, 2004). Both the parasympathetic and sympathetic nervous systems innervate the endocrine pancreas by directly innervating the islet cells in order to adjust glucose homeostasis with food intake (Teff, 2011; Thorens, 2011; Rodriguez-Diaz et al., 2012). It is thought that this parasympathetic-sympathetic balance works to properly regulate the pancreas for normal functioning.

Parasympathetic preganglionic neurons involved in innervating the pancreas reside in the dorsal vagal nucleus within the brainstem. From here, parasympathetic preganglionic neuron axons travel out of the CNS to synapse upon parasympathetic postganglionic neurons located in the pancreatic ganglia. Parasympathetic postganglionic neurons directly innervate the pancreas (Rodriguez-Diaz et al., 2012).

The sympathetic circuit responsible for controlling the pancreas is similar to that for the liver, gallbladder, and bile ducts. SPNs located in the T6-L2 IML send axons out through the splanchnic nerve to the celiac ganglion. The sympathetic postganglionic neurons within the celiac ganglia innervate the pancreas (Sharkey and Williams, 1983; Renehan et al., 1995; Won et al., 1998).

Spinal cord injury at or above T6-L2 would disrupt the neural control of the pancreas, causing dysfunction of the organ. One such consequence is increased occurrence of pancreatitis (Nobel et al., 2002; Pirolla et al., 2014; Ho et al., 2021). Pancreatitis, a life-threatening disease that needs to be assessed immediately, is an increased inflammatory response with increased serum levels of pancreatic enzymes, such as p-amylase and lipase, which can cause corrosion of the pancreatic parenchyma (Nobel et al., 2002; Pirolla et al., 2014). This corrosion can result in devastating damage inflammation and/or necrosis that can then spread to other organs, causing their failure (Pirolla et al., 2014; Schepers et al., 2019).

It has been suggested that pancreatitis could also be caused be a disrupted balance of the sympathetic-parasympathetic control of the sphincter of Oddi, a smooth muscle that is located at the end of the gallbladder and pancreatic ducts to allow for bile and pancreatic juice to flow into the duodenum of the small intestine (Carey et al., 1977). The sphincter of Oddi receives innervation from both the sympathetic nervous system *via* the superior mesenteric ganglion and the parasympathetic nervous system *via* the vagus nerve. Increased cholinergic activity produces spasms of the sphincter of Oddi (Carey et al., 1977; Novaes et al., 1982). These spasms result in retention of pancreatic enzymes, increasing the risk of pancreatitis (Carey et al., 1977; Novaes et al., 1982; Chen et al., 2000). Following SCI, increased sympathetic nervous system activity produces spasms of the sphincter of Oddi and stasis of pancreatic secretions.

Inside the pancreas are clusters of beta cells called islets that are responsible for production of the hormones insulin and glucagon (Rodriguez-Diaz and Caicedo, 2014). These hormones are delivered to the liver to maintain glucose homeostasis (Rodriguez-Diaz and Caicedo, 2014). Pancreatic islets are directly innervated by the sympathetic nervous system. While the islet cells can function without any autonomic innervation (Brodows et al., 1976; Corrall and Frier, 1981; Rodriguez-Diaz and Caicedo, 2014), it is clear that sympathetic innervation plays a role in normal pancreatic function. Loss of sympathetic input to the islet cells is associated with impaired glucagon response and the onset of diabetes (Mei et al., 2002; Taborsky et al., 2009). Thus, it makes sense that a SCI that perturbs sympathetic regulation would disrupt glucose homeostasis. Thus, SCI can increase risk of diabetes through dysfunction of multiple organs, including the liver, as described above (Bauman and Spungen, 2001; Lee et al., 2005), and the pancreas (Cheng et al., 2020).

### 4.8. Gastrointestinal tract

The gastrointestinal (GI) tract is comprised of all the major organs of the digestive system. After food is swallowed, it goes through the throat and down the esophagus to the stomach. The stomach serves as both a reservoir for food as well as a digestion point. Once food reaches the stomach, the stomach contracts and produces digestive enzymes to help break down the food prior to passing to the small intestine and large intestines. The lower GI tract consists of the colon and the rectum which are the final two digestive processes where reabsorption, storage, and elimination happens (Holmes and Blanke, 2019).

Some parts of the GI tract, such as the intestines, can mostly control itself with the enteric nervous system (Browning and Travagli, 2014). The enteric nervous system is embedded in the wall of the GI tract. This nervous system allows for the GI system to control itself independently of the CNS. The enteric nervous system is made up of the myenteric plexus and submucosal plexus and includes sensory, motor, and interneurons to work to coordinate gut motility and secretions (Chung and Emmanuel, 2006). The enteric nervous system contains complete reflex circuits consisting of sensory neurons that sense changes within the digestive system, a local circuit of neurons that is responsible for integrating this information, and motor neurons that regulate activity of the muscles within the GI tract that mediate gut movement, secretions of digestive enzymes, mucus, and stomach acid.

Although the enteric nervous system has been shown to be able to work independently of the CNS, it still communicates heavily with the two autonomic nervous system branches to regulate GI functions (Bornstein et al., 1988; Chung and Emmanuel, 2006; Browning and Travagli, 2014). Both the parasympathetic and sympathetic postganglionic neurons synapse directly onto neurons within the enteric nervous system (*via* the vagus nerve and the pelvic nerves) to regulate their activity. The two branches of the autonomic nervous system provide a connection between the enteric nervous system of the gut and the CNS. As mentioned previously, the parasympathetic system is referred to the “rest and digest” system. Therefore, it makes sense that the parasympathetic system activates the GI tract for digestion by stimulating GI secretion and motor activity of GI muscles. Oppositely, the sympathetic nervous system works to inhibit release of digestive secretions, inhibit contraction of GI sphincters, and regulates blood flow to the GI tract *via* vasoconstriction (Bornstein et al., 1988; Browning and Travagli, 2014).

Normal function of the stomach relies more heavily on autonomic control in addition to the enteric nervous system. Sympathetic innervation of the stomach is similar to the liver, gallbladder, and pancreas. SPNs in T6 to T9 project axons out *via* the greater splanchnic nerve and synapse on the postganglionic cells in the celiac ganglion. Those sympathetic postganglionic neurons innervate the stomach (Chung and Emmanuel, 2006; Browning and Travagli, 2014).

Sympathetic function is also important for function of the ascending colon and the small and large intestine. Axons from SPNs in T9 to T12 travel out of the cord *via* the lesser splanchnic nerve to the and synapse onto postganglionic neurons in the superior mesenteric ganglion. These neurons innervate the ascending colon and the large and small intestine (Chung and Emmanuel, 2006; Browning and Travagli, 2014). The descending colon and the rectum receive input from the postganglionic neurons in the inferior mesenteric ganglion. These neurons receive input *via* the lumbar splanchnic nerve upon from SPNs in T12 to L3 (Chung and Emmanuel, 2006; Browning and Travagli, 2014). Thus, SCI at or above L3 could result in dysfunction of the GI tract.

Gastrointestinal tract complications have been shown to be more frequent in individuals with SCI compared to uninjured individuals (Gore et al., 1981; Chen et al., 2004; Holmes and Blanke, 2019). SCI individuals are more prone to experience heart burn, chest pain, and hiatus hernias where the upper stomach pushes through the muscle in between the abdomen and diaphragm creating chest pain, and inflammation of the esophagus (esophagitis), which could all be due to an increase of gastroesophageal reflux disease (GERD) following SCI (Stinneford et al., 1993; Singh and Triadafilopoulos, 2000; Silva et al., 2008). Gastroesophageal reflux is back flow of stomach acid or bile flowing back through the esophagus, which can irritate the esophagus and producing heartburn and/or acid reflux. The increased prevalence of GERD within the SCI population increases the risk of esophagitis following SCI.

Neurogenic bowel is a common colonic dysfunction following a SCI at or above L3 SCI. This is associated with symptoms like constipation, abnormal elimination reflexes, and reduced contractions of the colon (Lynch et al., 2001; Coggrave et al., 2014). This decreased intestinal movement can result in lack of blood flow and tissue death of the intestines, gastric dilation or twisted stomach, ulcers of the stomach lining or small intestines have all been increased acutely following SCI (Gore et al., 1981). Other symptoms include incontinence, abdominal pain, abnormal gastric activity, vomiting, nausea, peptic ulcers, diarrhea, rectal bleeding, hemorrhoids, and fecal impactions have all been reported to be higher in individuals suffering from SCI (Gore et al., 1981; Han et al., 1998; Vallès et al., 2006; Ebert, 2012).

Individuals with a mid-thoracic or higher SCI have increased gastrin section and decreased gastrointestinal emptying and motility (Bowen et al., 1974; Kewalramani, 1979; Berlly and Wilmot, 1984; Fynne et al., 2012). This slower GI emptying and movement and increased gastrin secretion could lead to gastroduodenal bleeding or Cushing’s ulcer which has shown to be increased in individuals with an SCI at T5 or higher (Cushing, 1932; Bowen et al., 1974; Kewalramani, 1979; Berlly and Wilmot, 1984).

As previously mentioned, the autonomic nervous system is a link between the bidirectional gut-brain axis. Recently, the gut microbiota, microorganisms residing within digestive tracts, have been shown to influence gut-brain axis (Carabotti et al., 2015). The gut microbiota has been of increasing interest to the SCI field since the sympathetic nervous system provides a link between the gut and the brain and has been shown to be influenced by the gut microbiota. Moreover, the gut microbiota have been shown to play a critical role in the maintenance of homeostatic processes (Gilbert et al., 2018). The development of neurogenic bowel post-SCI is associated with increased gut dysbiosis, a disrupted balance of the microbiota residing within the GI tract (Carding et al., 2015), with an increase in pro-inflammatory bacteria (Gungor et al., 2016; Kigerl et al., 2018; Bazzocchi et al., 2021). This increased prevalence of gut dysbiosis within the SCI population has been associated with GI disorders like irritable bowel syndrome (IBS) and inflammatory bowel diseases in the SCI community (Han et al., 2009; Tseng et al., 2017). Preclinically, gut dysbiosis has been shown to impair recovery following SCI and a diet rich in probiotics could serve as a potential therapeutic for sympathetic induced gut dysbiosis following SCI (Kigerl et al., 2016). As it is becoming more apparent that the microbiome shapes many aspects of overall health, it is very important to increase understanding of the implications of SCI-induced gut dysbiosis.

### 4.9. Spleen

The spleen is a part of the lymphatic system that is located between the diaphragm and the stomach within the abdomen. The spleen serves many necessary functions. It removes damaged red blood cells and controls the white to red blood cell balance. The spleen also plays a key role in the body’s immune defense by fighting invading bacteria *via* antibody production (Bronte and Pittet, 2013; Noble et al., 2018). The spleen stores leukocytes, immune cells that work to fight infections within the body, including neutrophils, eosinophils, and basophils, monocytes, and T and B cells. When the body is fighting off infections, the number of leukocytes increase and is referred to leukocytosis. Because of its imperative role in immune function, people without a spleen, or with a malfunctioning one, are at greater risk for infections (Madden et al., 1997; Davidson and Wall, 2001). Innervation of the spleen *via* the sympathetic chain ganglia increases throughout development, suggesting the need for this vital organ throughout life (Madden et al., 1997).

Sympathetic preganglionic neurons that play a role in regulating spleen function are located in the IML of T4–T12 (Anderson et al., 1989; Nance and Burns, 1989; Strack and Loewy, 1990; Cano et al., 2001). They project out of the spinal cord *via* the greater splanchnic nerve to the celiac ganglion, where the SPNs form synapses on the postganglionic neurons that then directly innervate the spleen (Felten et al., 1985; Straub, 2004; Nance and Sanders, 2007; Bratton et al., 2012). The parasympathetic nervous system does not innervate the spleen (Bellinger et al., 1993; Bratton et al., 2012).

Damage to the sympathetic nervous system *via* SCI impact’s splenic function. Immune suppression following SCI is commonly reported and is likely due to disrupted modulation of sympathetic innervation of the spleen (reviewed by Noble et al., 2018). Specifically, individuals with an SCI above sympathetic innervation of the spleen (T3 or higher) have increased risk of infections due to an immune suppressive response (Brommer et al., 2016). Preclinical SCI models suggest that increased activation of the sympathetic nervous system after high-level SCI results in splenic atrophy and leukopenia, low white blood cell count within the spleen, to contribute to immunodeficiency (Zhang et al., 2013; Ueno et al., 2016; Mironets et al., 2018, 2020). As a result, high-level SCI results in impaired ability to fight off bacterial and viral infections, leading to increased mortality within the SCI community (Bracchi-Ricard et al., 2016; Mironets et al., 2020). Preclinical data shows that this dysfunction has been associated with impaired T-cell function (Zha et al., 2014; Bracchi-Ricard et al., 2016). Interestingly, treatment prior to SCI with memory T cells were shown to be effective in the weeks following SCI for protection against infections (Norden et al., 2019).

Splenic dysfunction after SCI has been linked to accumulations of norepinephrine and glucocorticoids (Popovich et al., 2001; Lucin et al., 2007; Zhang et al., 2013). The hypothalamic-pituitary-adrenal axis (HPA axis) is responsible for glucocorticoid production while the sympathetic nervous system produces norepinephrine. Normally, the HPA axis and the sympathetic nervous system work in concert to facilitate proper immune functioning. Following SCI, the HPA axis is activated and the control over the sympathetic system is impaired further supporting the accumulations of norepinephrine and glucocorticoids (Lucin et al., 2007; Zhang et al., 2013). After SCI at T3, levels of both cortisol and norepinephrine are elevated and there is impaired antibody synthesis and splenocyte cell death (Lucin et al., 2007; Zhang et al., 2013; Prüss et al., 2017). This is not the case after a lower thoracic injury (T9) that spares more descending input to the spinal sympathetic circuit; levels of cortisol are increased but levels of norepinephrine and antibody synthesis are similar compared to uninjured controls (Lucin et al., 2007; Zhang et al., 2013; Prüss et al., 2017).

Interestingly, treatments that decrease plasticity of the sympathetic nervous system after SCI, such as gabapentin and the TNFα inhibitor XPro1595 and attenuate sympathetic hyperreflexia have also been shown to reduce SCI-associated splenic atrophy and loss of leukocytes (Mironets et al., 2018, 2020; Brennan et al., 2021).

### 4.10. Adrenal gland

The adrenal gland secretes hormones to serve in many essential functions like responding to stress, regulation of metabolism, blood pressure, and immune function. An adrenal gland sits on top of each kidney and the internal medulla is surrounded by the outer cortex. Although these two components seemingly serve two distinct functions, they are largely interconnected (Bornstein et al., 1991). The adrenal cortex produces steroid hormones like androgens and estrogens to regulate sexual function and aldosterone to regulate blood pressure by regulating the amount of salt and water within the body. The adrenal cortex also produces cortisol *via* the HPA axis in response to stress (Gavrilovic and Dronjak, 2005). The adrenal medulla contains Chromaffin cells that secrete catecholamines, like epinephrine and norepinephrine, needed for the sympathetic fight-or-flight response (Bornstein et al., 1991). Because of this, the adrenal medulla is highly interconnected with the sympathetic nervous system and relies on it for proper functioning (Goldstein and Kopin, 2008).

Sympathetic innervation of the adrenal gland is slightly different compared to the previously discussed organ systems. SPNs innervating the adrenal gland reside in the IML in the spinal cord at T9–T10. However, instead of leaving the spinal cord and entering a ganglion to synapse on the postganglionic neurons, SPNs travel out of the spinal cord *via* the lesser splanchnic nerve and synapse directly onto the adrenal gland (Kesse et al., 1988; Parker et al., 1993). It has been suggested that the adrenal medulla serves as a modified sympathetic ganglion and Chromaffin cells could serve as the postganglionic sympathetic neurons in this system.

Abnormal functioning of the HPA axis is thought to result in adrenal insufficiency and dysfunction of the adrenal gland, causing the body to improperly deal with stressors, such as a SCI itself (Lecamwasam et al., 2004). Adrenal insufficiency has been shown to be increased in SCI individuals compared to non-injured individuals (Garcia-Zozaya, 2006; Pastrana et al., 2012). Adrenal insufficiency can surface as hypotension, hyperkalemia, and hyponatremia (Lecamwasam et al., 2004; Garcia-Zozaya, 2006; Pastrana et al., 2012). While disruption of the HPA axis and the sympathetic nervous system plays a role in SCI-induced suppression of the immune system, as discussed above, SCI may also directly increase activation of the adrenal gland to increase cortisol levels, independent of the HPA axis, as an additional mechanism for SCI-induced suppression of the immune system (Prüss et al., 2017).

### 4.11. Urinary system

The urinary system (kidneys, ureters, bladder, and the urethra) serves many essential functions. It works to remove waste and regulate vitamins and minerals within the body. The urinary system is also involved in blood pressure regulation. The urinary system requires the involuntary control of the autonomic nervous system. Because of this, damage to the sympathetic nervous system disrupts supraspinal control of the urinary system resulting in organ dysfunction. The upper urinary tract consists of the kidneys and the ureters while the lower urinary tract consists of the bladder and the urethra. The kidneys are connected to the bladder by tubes called ureters. Once the kidneys filter out the fluids, urine flows through the ureters to the bladder where it is stored until voiding. Usually, the ureters serve as a one-way system and urine is unable to back flow into the kidneys. However, if the bladder becomes too full or there are problems within this system, such as abnormal sympathetic nervous system function, urine can backflow and cause both bladder and kidney problems.

#### 4.11.1. Kidney

The kidneys function to filter blood and discard the waste and regulate the homeostasis of chemicals and minerals within the body. The kidneys also release hormones that help regulate blood pressure, produce red blood cells, and create healthy bones. They require input and regulation from the sympathetic nervous system to function properly. SPNs regulating kidney function are in the IML of T11-L1 (Ferguson et al., 1986; Huang et al., 2002). Axons from these SPNs leave the spinal cord and travel to the celiac ganglion, where they synapse with postganglionic sympathetic neurons that directly innervate each of the kidneys. Thus, SCI that results in dysfunction of the sympathetic nervous system can impact the kidneys.

Kidney problems are prevalent following SCI and are closely related to bladder and lower urinary tract problems. SCI at or above T11-L1 results in loss of descending inhibitory control over the sympathetic nervous system that innervates the kidneys. It has been found that individuals with SCI have increased prevalence of renal calculi or kidney stones (Welk et al., 2017). This increased risk of kidney stone development is thought to be due to the dysfunction of the mineral homeostasis function of the kidneys, therefore increasing calcium levels that then crystalize and form kidney stones (Naftchi et al., 1980; Shunmugavel et al., 2010). If left untreated, kidney stones can increase the risk of kidney failure and kidney atrophy (Constantinople et al., 1979). Similarly, loss of sympathetic regulation of the kidneys disrupts the function of regulating chemicals and minerals within the body. Because of this, hyponatremia, an abnormal decrease in blood sodium level, has been shown to be increased in patients with high level SCI (Furlan and Fehlings, 2009). Hyponatremia can also increase the risk of hypotension (Furlan and Fehlings, 2009). Similarly, oxidative stress following injury produces renal inflammation which has been shown to be a large contributor to renal crystal formation (Khan, 2005).

We recently found that high-level SCI dramatically alters kidney function. Because kidneys are highly vascularized, there is a need to protect this critical organ from fluctuations in systemic blood pressure. Renal autoregulation is a homeostatic mechanism that regulates the amount of blood flowing through the kidney. As systemic blood pressure increases, resistance of the renal vasculature increases to maintain a constant renal blood flow. Surprisingly, after chronic high-level SCI in rodents, renal autoregulation is completely abolished; there was virtually no change in renal vascular resistance in response to an increase in blood pressure, allowing more blood to flow unimpeded into the kidney. This loss of renal autoregulation was associated with injury to the kidney, as indicated by higher levels of neutrophil gelatinase-associated lipocalin, fibrosis, and inflammatory signaling than in animals with low-level SCI (Osei-Owusu et al., 2022).

#### 4.11.2. Bladder

**FIGURE 6:**
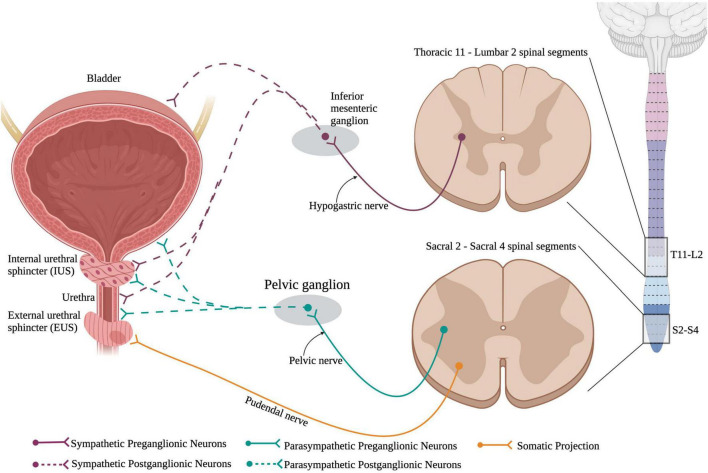
Neuronal control of the lower urinary tract (LUT). The lower urinary tract receives neuronal control from both branches of the autonomic nervous system and the somatic nervous system. Sympathetic control arises from sympathetic preganglionic neurons in thoracic segments 11 to lumbar segment 2 that extend axons out the hypogastric nerve and synapse onto sympathetic postganglionic neurons within the inferior mesenteric ganglion. These sympathetic postganglionic neurons innervate the bladder, IUS, and urethra. Parasympathetic preganglionic neurons in sacral spinal cord segments 2–4 project axons out of the cord *via* the pelvic nerve and synapse onto parasympathetic postganglionic neurons within the pelvic ganglion. These postganglionic neurons directly innervate the bladder, IUS, and urethra. Interestingly, the external urethral sphincter is the only muscle in the LUT that is controlled *via* the somatic nervous system. This allows for voluntary control over urinary functions. (Created using www.biorender.com).

The bladder is responsible for storage of urine and periodically voiding urine, also known as micturition, when the bladder becomes too full, and a threshold is reached. Normal urinary storage and voiding requires the integration of autonomic and somatic controls. The two continuous activities of filling and voiding are coordinated through contraction and relaxations of the bladder detrusor, urethra, and urethral sphincter muscles which are under high autonomic control (Figure 6). The external urethral sphincter is a striated muscle that is controlled by the somatic nervous system.

**FIGURE 7:**
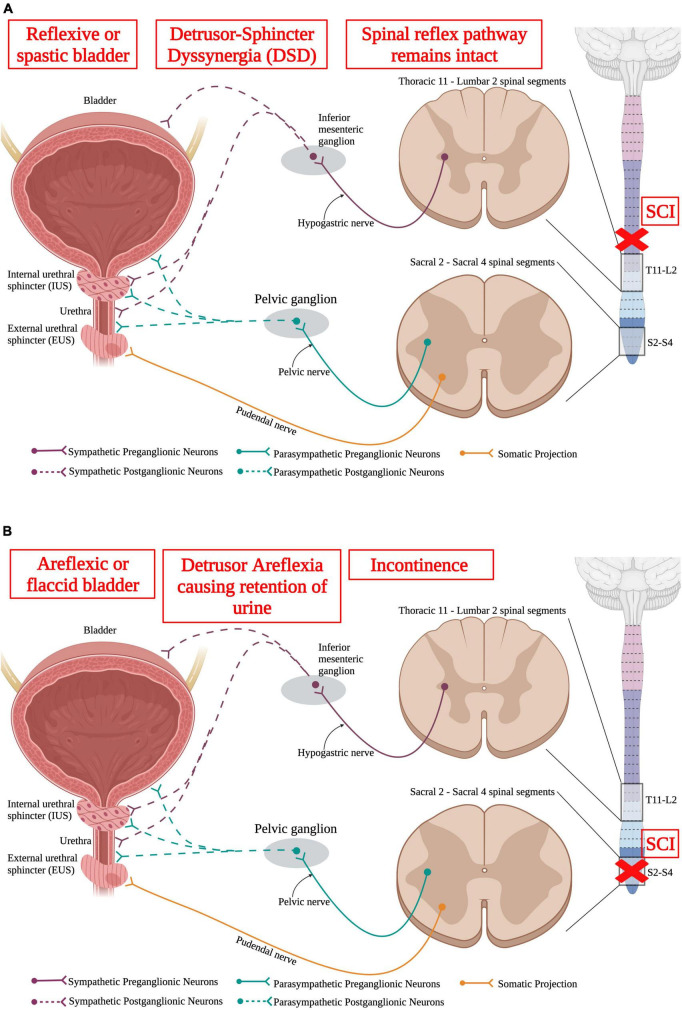
Spinal cord injury results in lower urinary tract (LUT) dysfunction. **(A)** An upper motor neuron injury is when there is no direct damage of the neurons that innervate the bladder, leaving the spinal reflex pathway intact. An upper motor neuron injury results in a reflexive or spastic bladder and detrusor-sphincter dyssynergia (DSD), where the detrusor and sphincter muscles abnormally contract simultaneously, resulting in inefficient voiding. **(B)** Lower motor neuron injury results in damage to the neurons that directly innervate the bladder. A lower motor neuron injury produces an areflexic or flaccid bladder where the detrusor muscle does not contract. This results in retention of urine in the bladder that makes the bladder become over-stretched. (Created using www.biorender.com).

Storage and voiding of urine require both sympathetic and parasympathetic pathways. Supraspinal control from Barrington’s nucleus and the periaqueductal gray region in the brainstem sends projection down to both the sympathetic circuit in T11-L2 and the parasympathetic circuit in S2–S4. SPNs in T11-L2 extend out of the ventral root of the spinal cord and form the hypogastric nerve to synapse onto sympathetic postganglionic neurons in the inferior mesenteric ganglion that directly innervate the bladder detrusor and urethra muscles (Hou and Rabchevsky, 2014). Parasympathetic preganglionic neurons reside in the sacral spinal cord (S2–S4) and project out to synapse on parasympathetic postganglionic neurons. Therefore, SCI at or above the lower thoracic segments would affect the sympathetic nervous system and disrupt urinary function (Figure 7).

In infants, control to void or store urine is regulated by the involuntary autonomic nervous system. This is seen when infants are unable to control the storage of their urine and void spontaneously. However, as humans mature, they obtain conscious control over the bladder, deciding whether to void or continue storing urine. Control of the bladder works somewhat like a switch. As the bladder fills, the external urethral sphincter is contracted while the detrusor muscle remains relaxed. Once the bladder becomes full, the switch is flipped from storage to voiding where the sphincter relaxes while the detrusor contracts, allowing for micturition. Both functions of storing urine and micturition require its unique sets of neural controls, such as autonomic and somatic circuits within the spinal cord that communicate with supraspinal areas for further regulation (Fowler et al., 2008). Within the autonomic nervous system control, the sympathetic nervous system works to inhibit detrusor contraction to facilitate the ability to store urine. As the bladder fills and reaches a particular threshold, the parasympathetic pathway then takes over to contract the detrusor muscle and expel urine from the bladder.

Normally, the detrusor muscle contracts as the sphincter muscle relaxes to void the bladder. However, after SCI, the detrusor and sphincter have been shown to contract simultaneously resulting in detrusor-sphincter dyssynergia causing insufficient voiding (Chang et al., 2000; Fowler et al., 2008). These urinary problems, also known as neurogenic bladder, refers to the abnormal ability for the bladder to fill, store, and void urine efficiently, is very common following SCI (Ruutu and Lehtonen, 1984; Chao et al., 1993; Waites et al., 1993; Chen et al., 1999; Lawrenson et al., 2001). Because of this, urine can sit in the urinary tract longer than necessary and exposes the urinary system to bacteria, which increases the chances of infection within SCI individuals in both the upper and lower urinary tracts and can ultimately lead to renal failure (Ruutu and Lehtonen, 1984; Chao et al., 1993; Lawrenson et al., 2001; Welk et al., 2017). Thus, SCI patients have higher rates of urinary tract infections that, if left untreated, could be life threatening (Waites et al., 1993; Welk et al., 2017).

Neuroplasticity after SCI is thought to contribute to aberrant bladder function. Specifically, increased levels of NGF after SCI causes neurons to undergo morphological and physiological changes such as bladder enlargement, increased CGRP sprouting, and tyrosine hydroxylase-positive (TH)^+^ sympathetic fibers (Kruse et al., 1995; Yoshimura and de Groat, 1997; Schnegelsberg et al., 2010). These changes have been shown to lead to bladder hyperactivity, detrusor overactivity, and increased detrusor-sphincter dyssynergia (Seki et al., 2002, 2004; Vizzard, 2006). Treatment with anti-NGF antibodies to prevent some of this maladaptive plasticity in SCI models has been shown as a possible treatment to decrease detrusor overactivity and detrusor-sphincter dyssynergia (Seki et al., 2002, 2004; Vizzard, 2006).

Following SCI, the bladder is initially areflexic. However, over time, it regains partial functional recovery due to plasticity. In an intact system, small myelinated (Aδ) afferents relay information directly from the bladder to the spinal cord and are responsible for mediating micturition. However, following SCI, unmyelinated (C) fibers have been shown to sprout and contribute more to micturition (Kruse et al., 1995; de Groat and Yoshimura, 2006, 2012). Specifically, C-fiber soma size increases after SCI (Kruse et al., 1995; Yoshimura and de Groat, 1997), and they also have a lower activation threshold (Yoshimura and de Groat, 1997). Similarly, it has been found that TH^+^ neurons reside in the lumbosacral spinal cord further supporting their involvement in the spinal bladder reflex. Interestingly, following SCI, there is an increase in TH^+^ fibers which regulate the partial recovering of micturition (Hou et al., 2016; Qiao et al., 2021). This is further supported by work that shows depleting TH^+^ fibers in the lumbosacral spinal cord results in a decrease of bladder contractions and the ability to void urine (Hou et al., 2016). The partial recovery of micturition with the spinal bladder reflex, depicts an example of the benefits of intraspinal plasticity following SCI.

Interestingly, neuromodulation *via* electrical stimulation is a promising means to improve bladder function after SCI. Epidural stimulation of the lumbosacral spinal cord was shown to improve bladder function (e.g., voiding) after SCI (Herrity et al., 2018; Walter et al., 2018). It is thought that epidural stimulation increases activation of severed spinal circuits below the level of injury to work to restore function following SCI (Herrity et al., 2018; Walter et al., 2018). Specifically, epidural stimulation in rodents with SCI elicits voiding within seconds of onset of stimulation (Gad et al., 2014; Hoey et al., 2021). These preclinical data showed promising translational effects for epidural stimulation to be examined in humans. Epidural stimulation was shown to attenuate neurogenic bladder in humans (Walter et al., 2018) and also improve voiding function *via* modulating detrusor contraction and subsequent external urethral sphincter relaxation in SCI people (Herrity et al., 2018; Walter et al., 2018).

Stimulation of the spinal cord is not the only means to improve bladder function. Stimulation of the pudendal nerve in the pelvic region also improves voiding function and increased bladder contractions in chronic SCI cats (Tai et al., 2007). Specifically, this work showed that during bladder filling, pudendal nerve stimulation inhibited reflexive voiding and increased bladder capacity (Tai et al., 2007). Furthermore, pudendal nerve stimulation also improved voiding efficiency following SCI (Naderi and Safarinejad, 2003). Overall, neuromodulation *via* epidural or pudendal nerve stimulation is a promising therapeutic treatment to improve bladder function for people living with SCI.

### 4.12. Reproductive system

Males and females have their own unique reproductive organs responsible for producing sperm or egg cells, transporting these cells, and facilitate proper fertilization to produce offspring. The male reproductive organs consist of the testicles, the vas deferens, prostate gland, and the penis. The female reproductive organs consist of ovaries, fallopian tubes, and the vagina. The reproductive organs not only serve for producing offspring, but they are also involved in sexual function of both males and females. Interestingly, individuals who sustained a SCI list regaining sexual function as a higher priority over restoring fertility (Anderson et al., 2007a), indicating how much sexual function impacts quality of life.

Control over the reproductive system and sexual function heavily involves both branches of the involuntary autonomic nervous system and the somatic nervous system. Although there are distinct differences between male and female reproductive and sexual functions, there are some similarities. For example, the autonomic nervous system regulates vascular dilation for penile or clitoral erection, regulates prostatic or vaginal secretions, and regulates smooth muscle contractions in both the penis and vagina during erection and orgasm.

For the reproductive system, SPNs located in the IML of T12-L2 project out of the spinal cord to form the hypogastric nerve and synapse onto sympathetic postganglionic neurons in the prevertebral ganglia. These neurons directly innervate the reproductive organs in both males and females (Hancock and Peveto, 1979; Jänig and McLachlan, 1987). Therefore, SCI at or above this level cause abnormalities of reproductive and sexual function.

#### 4.12.1. Male

Penile erection requires a balance of the parasympathetic and sympathetic systems. Although the role of the sympathetic nervous system on penile erection is largely debated, generally the parasympathetic system serves a “pro-erectile” function whereas the sympathetic system serves a “anti-erectile” function (Diederichs et al., 1991; Giuliano and Rampin, 2000; Steers, 2000). However, following SCI, the parasympathetic system and local spinal reflex pathways that can elicit short-lived reflexive erections remain intact (Bodner et al., 1987; Courtois et al., 1993). There are two types of erections, reflex and psychogenic. A reflex erection involves the spinal parasympathetic reflex pathway residing in the sacral spinal cord. Upon physical contact of the penis, sensory information is relayed to the sacral spinal cord that results in motor output and penile erection. In SCI individuals where the sacral spinal cord is not damaged, reflex erections can still happen. The sympathetic nervous system, on the other hand, has been shown to modulate psychogenic erections that are independent of genital stimulation but are instead produced by thoughts, sights, sounds, and fantasies (Courtois et al., 1993; Biering-Sørensen and Sønksen, 2001). After stimulation, supraspinal centers send messages to the SPNs in the spinal cord causing the penis to become erect. Thus, SCI at or above T11 disrupts supraspinal control and decreases psychogenic erections (Anderson et al., 2007b). Injury at this level spares the lumbosacral reflex circuit, however, and a reflexogenic erection can still occur. However, this type of erection is often not rigid enough for intercourse.

Ejaculation also requires a balance of the parasympathetic and sympathetic nervous systems. Ejaculation can be broken down into two different components, emission and expulsion (Newman et al., 1982). The emission phase requires high input from the sympathetic nervous system where it causes the vas deferens and bladder to contract to allow for semen to enter the urethra (Giuliano and Clement, 2005). The sympathetic nervous system is implicated in preventing backflow of semen into the bladder (Shafik and El-Sibai, 2000). The parasympathetic nervous system is involved in the expulsion phase where it causes the urethra to contract and expel semen to the environment (Carro-Juárez et al., 2003; Motofei and Rowland, 2005). SCI individuals have significantly lower rates of ejaculation compared to non-injured individuals, and sometimes even no ejaculation during orgasm, also known as anejaculation (Chéhensse et al., 2013). Additionally, ejaculation in males with an injury at or above T6, has been shown to induce an autonomic dysreflexia event previously mentioned in the cardiovascular section (Sheel et al., 2005).

Spinal cord injury produces problems with penile erectile dysfunction and abnormal ejaculation, which can contribute to low fertility rates in SCI individuals. However, SCI also can result in necrospermia, where the sperm is not viable, hypogonadism, where the testes are dysfunctional, or hypospermatogenesis, where there is a decrease in the amount of sperm within the semen (Mallidis et al., 1994; Elliott et al., 2000; Naderi and Safarinejad, 2003; Brown et al., 2006; Patki et al., 2008). One possible mechanism for this decreased sperm motility in SCI males could be due to increased levels of leukocytes and inflammatory cytokines within the seminal plasma following SCI (Trabulsi et al., 2002; Basu et al., 2004).

Erectile dysfunction after SCI often results in relationship concerns, increased stress, anxiety, and depression, and decreased self-esteem and confidence. Along with the many changes that come with SCI, erectile dysfunctions can further worsen the psychological effects many suffer after SCI.

#### 4.12.2. Female

Female sexual arousal and clitoral erection also requires input from both the parasympathetic and sympathetic nervous systems. As with males, female sexual arousal can be broken down into reflexive arousal, which involves the parasympathetic pathway, and psychogenic arousal, which involves the sympathetic pathway (Sipski et al., 1995). The parasympathetic pathway *via* the pelvic nerves in S2–S4 controls things like clitoral erection, vaginal lubrication, and vulvar swelling (Park et al., 1997; Giuliano et al., 2001). The SPNs of the sympathetic system in T10-L2 eventually forming the hypogastric nerve innervates the cervix and uterus and initiate rhythmic contractions of these muscles and facilitates sexual arousal.

Individuals with a SCI that disrupts the supraspinal control over the sympathetic nervous system may still display reflexive sexual arousal signs if the parasympathetic reflexive control remains intact (Meston and Gorzalka, 1995; Lorenz et al., 2012).

Spinal cord injury individuals have reported difficulty becoming both psychologically as well as physically aroused (Anderson et al., 2007c; Othman and Engkasan, 2011; Hajiaghababaei et al., 2014). This is partly due to sympathetic nervous system dysfunction and impaired psychogenetic sexual arousal after SCI. Additionally, SCI damage to the cord at T12 or higher has been shown to decrease vaginal lubrication due to interruption of sympathetic innervation and ensuing disruption of vaginal blood flow (Bérard, 1989; Forsythe and Horsewell, 2006; Anderson et al., 2007c; Hajiaghababaei et al., 2014). However, reflexive lubrication in women with SCI at or above T12 is still functional due to the sacral parasympathetic nervous system being preserved (Bérard, 1989).

Nevertheless, the ability to achieve orgasm does not appear to be impacted by SCI, regardless of level or completeness of injury but that the latency to complete orgasm is longer in women with SCI compared to uninjured individuals (Sipski et al., 2001). Moreover, SCI women have been shown to have normal menstrual cycles within the year of injury onset and are able to conceive and carry infants to full term (Cross et al., 1992; Reame, 1992; Atterbury and Groome, 1998).

## 5. Considerations for the future

Depending on the level of injury and the severity of the injury, SCI can detrimentally impact descending inhibitory supraspinal control over the sympathetic nervous system. This, along with the intraspinal plasticity that occurs following SCI, results in detrimental dysfunction of multiple organ systems. Therefore, individuals suffering with SCI are at greater risk for a plethora of diseases associated with these multi-organ dysfunctions, greatly affecting their quality-of-life. Thus, it is important for future studies to examine the effectiveness of potential therapeutics in improving not only motor and sensory function but also autonomic function, as mitigating SCI-induced organ dysfunction would greatly and broadly benefit those living with SCI.

Unfortunately, there are no current treatments to repair the damage to spinal cord after SCI. This is in part due to the lack of complete understanding of what factors limit repair after the CNS. However, this is a very active area of research. Cell-based treatments have significant promise in treating individuals living with SCI. Various cell types, such as mesenchymal stem cells (MSCs), neural progenitor cells (NPCs), Schwann cells, and induced pluripotent stem cells (iPSCs), have all been explored as potential therapies (Assinck et al., 2017). Cell-based transplantation methods are advantageous due to the fact that specific populations of cells can be selected for grafting. For instance, we recently showed that transplanting NPCs that contained serotonergic neurons – a population that normally modulates activity of the spinal sympathetic circuit – into a high-level SCI site increased the number of serotonergic fibers innervating the intermediolateral cell column below the SCI, improved cardiovascular regulation after SCI in a serotonin-dependent manner (Hou et al., 2020). Additionally, transplanted cells can be modified to secrete specific growth factors for neuroprotection, as wells a promote things like axon regeneration and synapse formation (Assinck et al., 2017). Similarly, peripheral nerve grafts can provide growth-supportive substrates that allow damaged axons to regenerate (Côté et al., 2011). These natural scaffolds may possibly serve as a means to restore innervation from the brain to neurons and circuits below the level of injury.

Recovery of autonomic function may not need a therapy focused on cell replacement or promoting axon regeneration that would reform circuits. As mentioned above, plasticity within the spinal cord after SCI contributes to sympathetic dysregulation. While the mechanisms underlying this plasticity are still being elucidated, several have been identified. This knowledge allows these mechanisms to be targeted for therapeutic effects.

For instance, as previously mentioned, treatment with gabapentin immediately following SCI reduces autonomic dysreflexia-induced immunosuppression, splenic atrophy, and maintained B and T cell levels post SCI (Brennan et al., 2021). Gabapentin binds to α2δ calcium subunits and is associated with decreased excitability of neuronal circuits, which is why it is often used to treat neuropathic pain following SCI. It has also been shown to decrease excitatory synaptogenesis (Eroglu et al., 2009; Risher and Eroglu, 2012; Risher et al., 2018). Acute treatment with low- and midrange-dose gabapentin after SCI have been shown to reduce the magnitude of experimentally induced autonomic dysreflexia (Rabchevsky et al., 2011, 2012; Eldahan et al., 2020; Brennan et al., 2021). Additionally, chronic treatment with a very high dose of gabapentin also reduced autonomic dysreflexia (Brennan et al., 2021). However, chronic treatment of a midrange dose of gabapentin was conversely showed to increase naturally occurring autonomic dysreflexia frequency (Eldahan et al., 2020). While the mechanism in which gabapentin is working to reduce SCI-induced autonomic dysreflexia is likely layered, one possible thought is that acute gabapentin treatment is working to prevent the formation of excitatory synapses by blocking TSP (Eroglu et al., 2009) as well as block secretion of intraspinal glutamatergic secretion. While these data provide interesting insights on gabapentin as a potential therapeutic for sympathetic dysregulation, additional studies on dosing and timing is needed to understand the full therapeutic potential of using gabapentin.

An additional potential treatment is XPro1595, a biologic that inhibits the pro-inflammatory cytokine TNFα. TNFα has been implicated with plasticity of the spinal sympathetic circuit, such as primary afferent sprouting and recruitment of interneurons into the circuit. Intrathecal administration of XPro1595 following complete high-level SCI attenuates the frequency and magnitude of autonomic dysreflexia events (Mironets et al., 2018, 2020). This was associated with decreased immunosuppression and detrimental remodeling and increased sensitivity of the peripheral vasculature to vasopressors normally observed after high-level SCI (Mironets et al., 2018, 2020). While this is promising, it there appears to be a finite therapeutic window for XPro1595. Starting administration 2 weeks after SCI does not affect autonomic dysreflexia (O’Reilly et al., 2021), indicating that starting treatment in the subacute timeframe would be needed.

High-level SCI increases levels of norepinephrine that act on β2-adrenergic receptors (β2AR) (Lucin et al., 2007; Zhang et al., 2013). In uninjured individuals, activation of SPNs results in release of norepinephrine and glucocorticoids from postganglionic neurons and the adrenal gland which, in turn, activate β2ARs and play a role in normal immune function. However, prolonged SPN activation following SCI ultimately leads to prolonged activation of β2ARs, resulting in an immunosuppressive state in the SCI population (Lucin et al., 2007; Nance and Sanders, 2007). Therefore, a possible therapeutic for the immunosuppression associated with high-level SCI could be utilizing β2AR blockers to prevent prolonged activation of β2ARs associated with an immunosuppressive state (Lucin et al., 2007; Zhang et al., 2013).

Neuromodulation *via* electrical stimulation is a non-pharmacological therapy that has been shown to improve functional recovery after SCI. Epidural electrical stimulation of the lower thoracic spinal cord after a high-level SCI attenuates extreme blood pressure fluctuations, bladder, and sexual dysfunctions normally observed after such an injury (Phillips et al., 2018; Walter et al., 2018; Darrow et al., 2019). Although epidural stimulation provides a variety of promising evidence for functional recovery following injury, a main downfall to epidural stimulation is that it requires a fairly invasive surgery in order to implant the electrodes on the dura of the spinal cord itself. Due to the invasive nature of epidural stimulation, researchers have begun to explore other, less invasive stimulation techniques such as transcutaneous stimulation. Similar to epidural stimulation, transcutaneous stimulation works to provide electrical currents to activate neuronal circuits. However, in the case of transcutaneous stimulation, the electrodes are placed on the skin of the injured individual rather than on the spinal cord itself. Transcutaneous stimulation has shown to also ameliorate cardiovascular dysfunction as seen by attenuating orthostatic hypotension normally seen in SCI individuals (Phillips et al., 2018), as well as improving bladder capacity and decreasing detrusor contractions following injury (Doherty et al., 2019).

Another type of stimulation being explored as a therapy for SCI individuals is vagus nerve stimulation. Stimulation of the vagus nerve after SCI in preclinical models decreases heart rate, however this decrease is only seen while the stimulation is happening (Sachdeva et al., 2020). Importantly, vagus nerve stimulation does not appear to exacerbate autonomic dysreflexia events, alluding to its safety as a possible therapeutic to improve autonomic function after SCI (Sachdeva et al., 2020). Interestingly, vagus nerve stimulation in a rodent SCI model reduces neuroinflammation, i.e., expression of pro-inflammatory cytokines while also increasing the expression of anti-inflammatory cytokines, after SCI (Chen et al., 2022), suggesting that vagus nerve stimulation has broad effects and any recovery may be through multiple mechanisms. Although the overall concept of neuromodulation as a therapy for a myriad of dysfunctions seen after SCI is very exciting and full of potential, the devil is in the details on how the electrical stimulation is delivered, as there are ultimately pros and cons to each type of electrical stimulation technique. Additionally, it is likely that neuromodulation will be needed to be used in conjunction with other therapeutic treatments to provide optimal treatment to individuals living with SCI.

Although the spinal cord research field is constantly developing new therapeutic strategies, it is not likely that one therapy will produce complete recovery in spinal cord injured individuals. Because of the complex, multifaceted nature of SCI, it is likely a combination of therapies targeting various aspects will be needed to provide the best possible chance of recovery for SCI individuals. It is imperative that the research community continue to increase our mechanistic understanding of the consequences of SCI in order to shed light on what therapies need to target in order to improve the overall health and quality of life for those living with SCI.

## Author contributions

MW prepared the first draft of the manuscript. MW and VT edited and revised the manuscript. Both authors contributed to the article and approved the submitted version.
